# Fumarate respiration of *Fasciola* flukes as a potential drug target

**DOI:** 10.3389/fcimb.2023.1302114

**Published:** 2024-01-25

**Authors:** Atsushi Tashibu, Daniel Ken Inaoka, Kimitoshi Sakamoto, Kenji Murakami, Ferdoush Zannatul, Kiyoshi Kita, Madoka Ichikawa-Seki

**Affiliations:** ^1^ Laboratory of Veterinary Parasitology, Faculty of Agriculture, Iwate University, Morioka, Japan; ^2^ Department of Molecular Infection Dynamics, Shionogi Global Infectious Diseases Division, Institute of Tropical Medicine (NEKKEN), Nagasaki University, Nagasaki, Japan; ^3^ School of Tropical Medicine and Global Health, Nagasaki University, Nagasaki, Japan; ^4^ Department of Biomedical Chemistry, Graduate School of Medicine, The University of Tokyo, Tokyo, Japan; ^5^ Department of Biochemistry and Molecular Biology, Faculty of Agriculture and Life Science, Hirosaki University, Hirosaki, Japan; ^6^ Laboratory of Veterinary Microbiology, Faculty of Agriculture, Iwate University, Morioka, Japan; ^7^ Department of Host-Defense Biochemistry, Institute of Tropical Medicine (NEKKEN), Nagasaki University, Nagasaki, Japan

**Keywords:** *Fasciola*, respiratory chain, drug target, fumarate respiration, adult, newly excysted juveniles

## Abstract

Fascioliasis is a neglected tropical zoonotic disease caused by liver flukes belonging to the genus *Fasciola*. The emergence of resistance to triclabendazole, the only World Health Organization-recommended drug for this disease, highlights the need for the development of new drugs. Helminths possess an anaerobic mitochondrial respiratory chain (fumarate respiration) which is considered a potential drug target. This study aimed to evaluate the occurrence of fumarate respiration in *Fasciola* flukes. We analyzed the properties of the respiratory chain of *Fasciola* flukes in both adults and newly excysted juveniles (NEJs). *Fasciola* flukes travel and mature through the stomach, bowel, and abdominal cavity to the liver, where oxygen levels gradually decline. High fumarate reductase activity was observed in the mitochondrial fraction of adult *Fasciola* flukes. Furthermore, rhodoquinone-10 (RQ_10_ Em’= −63 mV), a low-potential electron mediator used in fumarate respiration was found to be predominant in adults. In contrast, the activity of oxygen respiration was low in adults. Rotenone, atpenin A5, and ascochlorin, typical inhibitors of mitochondrial enzymes in complexes I, II, and III, respectively, inhibit the activity of each enzyme in the adult mitochondrial fraction. These inhibitors were then used for *in vitro* viability tests of NEJs. Under aerobic conditions, NEJs were killed by rotenone or ascochlorin, which inhibit aerobic respiration (complex I–III), whereas atpenin A5, which inhibits complex II involved in fumarate respiration, did not affect NEJs. Moreover, ubiquinone-10 (UQ_10_ Em’= +110 mV), which is used in oxidative respiration, was detected in NEJs, in addition to RQ_10_. In contrast, under anaerobic conditions, rotenone and atpenin A5, which inhibit fumarate respiration (complex I–II), were crucial for NEJs. These findings demonstrate that NEJs have active hybrid respiration, in which they can properly use both oxygen and fumarate respiration, depending on oxygen availability. Thus, fumarate respiration is a promising drug target for *Fasciola* flukes, because it plays an essential role in both adults and NEJs.

## Introduction

Fascioliasis, caused by liver flukes of the genus *Fasciola* has a considerable economic impact on the livestock industry. Global economic losses due to this disease are estimated to exceed 3.2 billion USD annually (Spithill, 1999). Human fascioliasis is a neglected tropical zoonotic disease that affects 2.4 million people in more than 70 countries worldwide, with 180 million people at risk of infection. No continent excluding Antarctica is free of fascioliasis, and human cases likely exist where animal cases have been reported (WHO, 2020).

Triclabendazole (TCZ) is the most commonly used drug for treating fascioliasis. Currently, this is the only WHO-recommended drug for this disease. TCZ is effective against the adult and larval stages of the parasite. However, resistance to TCZ has been reported not only in livestock but also in humans (Overend and Bowen, 1995; Moll et al., 2000; Duthaler et al., 2010; Kelley et al., 2016). Infection levels continue to increase with an increase in TCZ resistance (Kelley et al., 2016). However, the mechanism underlying drug resistance development has not yet been fully elucidated (Fairweather, 2005; Brennan et al., 2007; Fairweather, 2009; Fairweather, 2011; Hodgkinson et al., 2013). Currently, no effective vaccines are available (McManus and Dalton, 2006; Wedrychowicz et al., 2007), which highlights the need to develop new drugs with a distinct mode of action (MOA).

**Figure 1:**
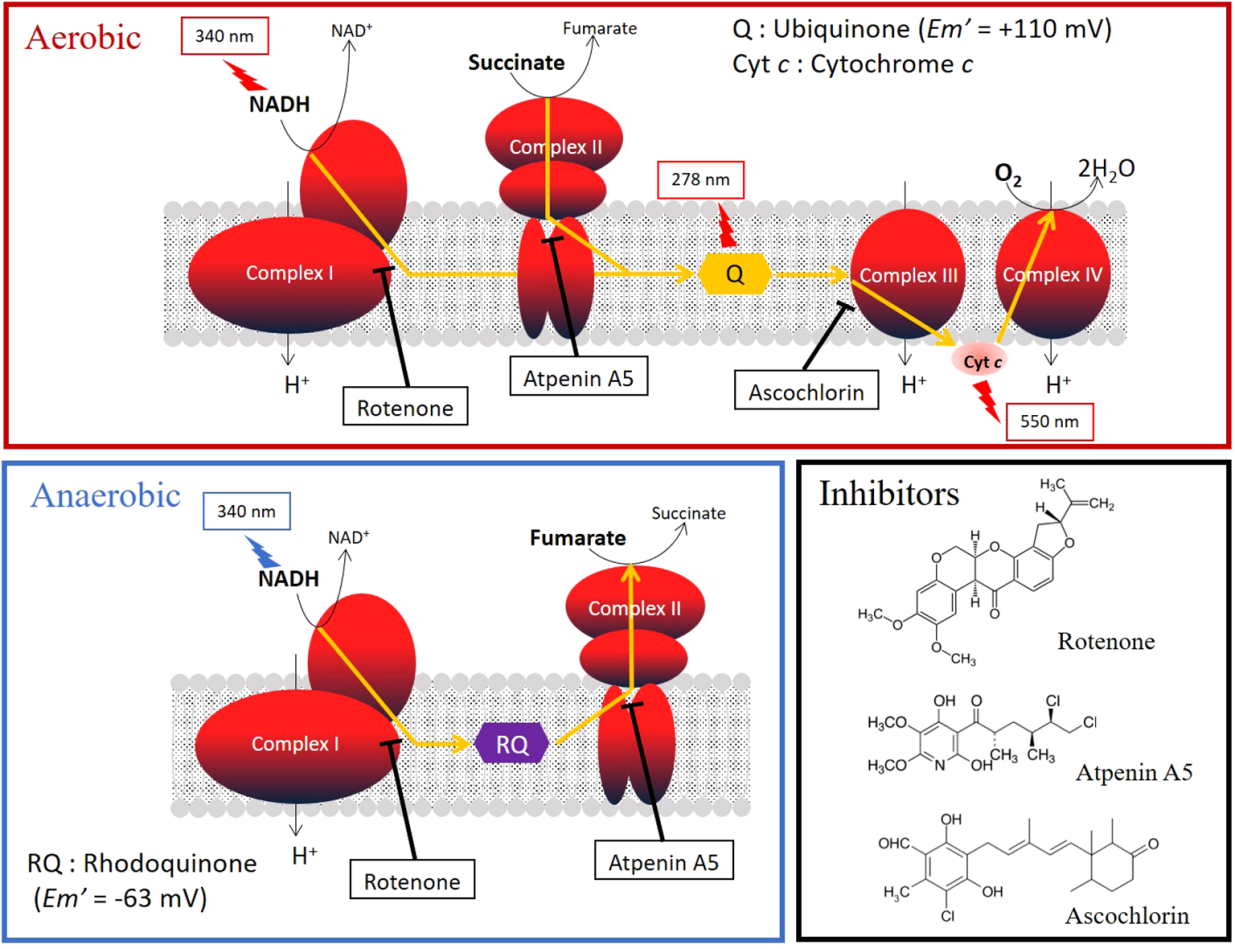
Oxygen (aerobic) and fumarate (anaerobic) respiration in the mitochondrial respiratory chain. The target sites and the structural formulas of typical inhibitors, rotenone, atpenin A5, and aschochlorin, which inhibit complexes I, II, and III, respectively, are shown. The absorbances of the substrates and quinones in the activity assays are shown in the figure.

Typically, the mitochondria in animals living in aerobic conditions require oxygen to function (Kita et al., 2003). Parasitic helminths exploit a variety of energy-transducing systems during adaptation to anaerobic habitats in their hosts (Kita et al., 2003). The NADH-fumarate reductase system is a part of the unique respiratory system of parasitic helminths and is the terminal step of the phosphoenolpyruvate carboxykinase-succinate pathway, which is found in many anaerobic organisms. The parasitic nematode *Ascaris suum* (Kita et al., 2003) and the cestode *Echinococcus multilocularis* (Matsumoto et al., 2008), which reside in the host’s small intestine, contain NADH-fumarate reductase to adapt to their microaerobic habitat. They use a low-potential rhodoquinone (RQ Em’= −63 mV) as an electron mediator instead of the ubiquinone (UQ Em’= +110 mV) used in mammalian cells (**Figure 1**). Electrons from NADH are accepted by the RQ via the NADH-RQ reductase activity of mitochondrial complex I and are then transferred to fumarate via the rhodoquinol-fumarate reductase activity of mitochondrial complex II. Electron transfer in complex I is coupled with the translocation of protons across the inner mitochondrial membrane, creating the electrochemical gradient required by complex V (ATP synthase) to provide ATP, even in the absence of oxygen (**Figure 1**). Mammalian mitochondria normally lack this system. Therefore, NADH-fumarate reductase has been proposed as a promising drug target for developing novel anthelmintics (Omura et al., 2001; Kita et al., 2003; Matsumoto et al., 2008; Enkai et al., 2023).

**Figure 2:**
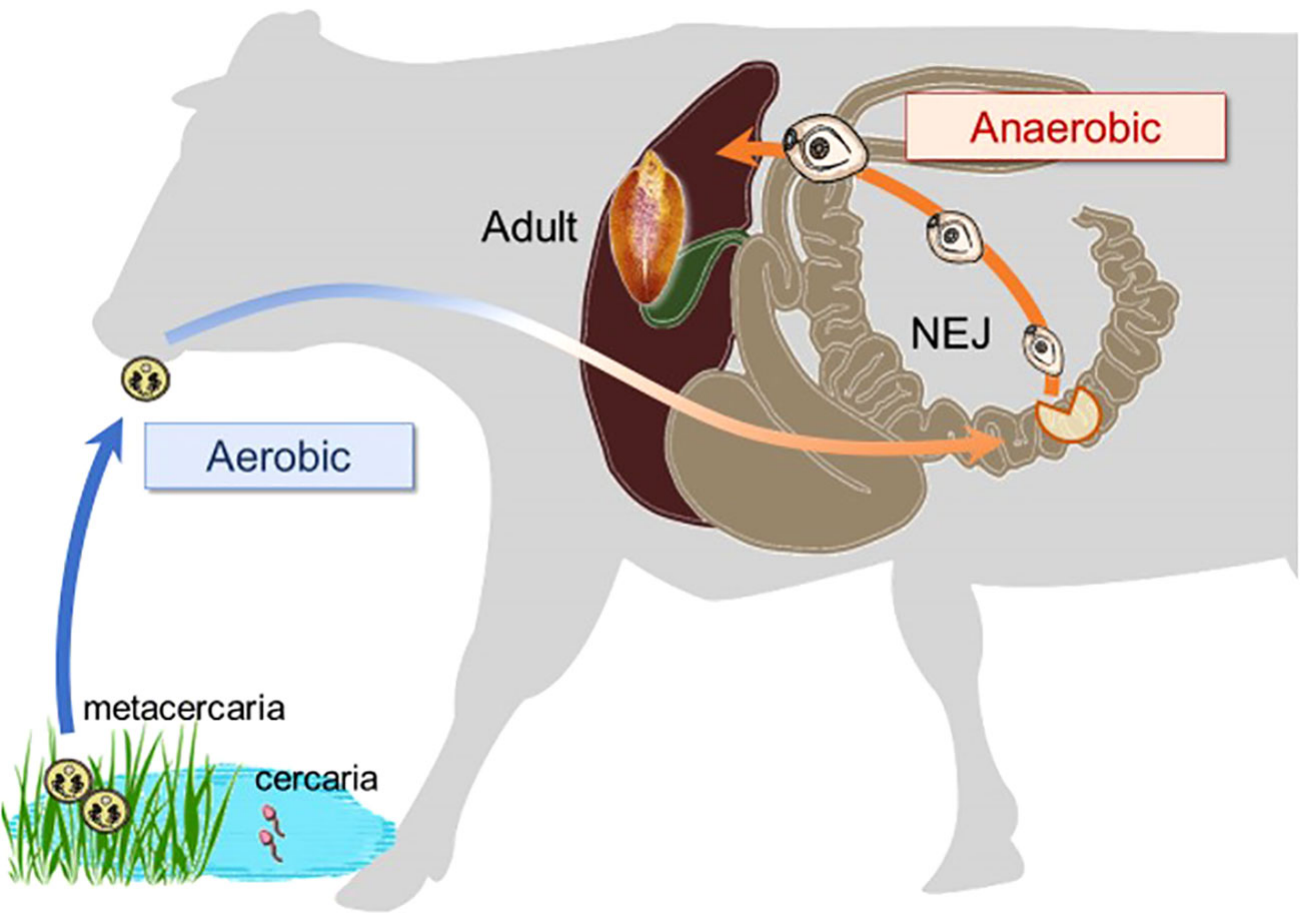
The changes in environmental oxygen concentration experienced during the life cycle of a *Fasciola* fluke, with a substantial shift occurring in the juvenile stage.

*Ascaris suum* successfully adapts to substantial reductions in oxygen availability during its life cycle by markedly altering its energy metabolism, from an aerobic pathway similar to mammalian hosts in the larval stage to a distinct anaerobic pathway in the adult stage (Iwata et al., 2008). Juvenile *Fasciola* flukes migrate through the stomach, intestine, and peritoneal cavity to the liver (**Figure 2**), where oxygen concentrations gradually decrease (Mas-Bargues et al., 2019). Consequently, the objective of this study was to characterize the mitochondrial respiratory chain of this trematode in both adults and newly excysted juveniles (NEJs) to evaluate its potential as a drug target. The respiratory systems of NEJs were analyzed using the viability assays employing specific inhibitors of respiratory chain enzymes because they are too small to obtain a mitochondrial fraction for biochemical studies.

## Materials and methods

### Preparation of adult flukes

A laboratory strain of wuh15-2 (*F. hepatica/gigantica* hybrid type), which was isolated from a cow in Wuhan, China, in 2007 (Ichikawa-Seki et al., 2017), was maintained in Wistar rats and *Orientogalba ollula* (syn. *Lymnaea ollula*) at the Laboratory of Veterinary Parasitology, Faculty of Agriculture, Iwate University, Japan. This strain was also used in a recent study (Sekii et al., 2023). To prepare the adult flukes, 10–20 metacercariae of the strain collected from intermediate host snails were orally infected with a male Wistar rat (6-week-old, SLC, Shizuoka, Japan). After more than two and a half months of infection, adult flukes were obtained from the bile ducts of the infected rats. The obtained flukes were washed with phosphate-buffered saline (PBS) and then rapidly frozen in liquid nitrogen and kept in a freezer (−80°C) until use. All animal experiments were conducted in compliance with the protocol approved by the Institutional Animal Care and Use Committee of Iwate University (A201836).

### Preparation of adult mitochondrial fractions

The mitochondrial fractions of adult *Fasciola* sp. were prepared according to a previously described protocol for adult *A. suum* mitochondria (Iwata et al., 2008). The frozen adult flukes (wet weight: 2.0g) were cut into small pieces and suspended in five volumes of mitochondrial preparation buffer (210 mM mannitol, 10 mM sucrose, 1 mM disodium EDTA, and 50 mM Tris-HCl pH 7.5) supplemented with 10 mM sodium malonate to stabilize complex II. The mixture was homogenized using a motor-driven glass/glass homogenizer with 15 strokes. The homogenate was diluted with the mitochondrial preparation buffer up to 10 times the volume of the original sediment and then centrifuged at 800 × g at 4°C for 10 min to precipitate any unbroken tissue and cell nuclei. The supernatant was then centrifuged at 8,000 × g at 4°C for 10 min to obtain the mitochondrial pellet. The pellet was resuspended in mitochondrial preparation buffer without malonate and centrifuged at 12,000 × g at 4°C for 10 min. The resulting mitochondrial fraction was resuspended in a mitochondrial preparation buffer without malonate. Protein concentration was determined according to the method of Lowry et al. (1951), using bovine serum albumin as a standard (Lowry et al., 1951).

### Characterization of respiratory chain activities of adult mitochondrial fractions

The enzyme activity assays and inhibitor effects were essentially performed in accordance with previous reports with slight modifications (Omura et al., 2001; Matsumoto et al., 2008; Enkai et al., 2023). Assays utilizing the adult mitochondrial fractions were carried out in a 1 mL reaction mixture at 25°C. The final mitochondrial protein concentration was 42.6 μg/mL of reaction mixture in each assay. The mean of triplicate assays was calculated to determine the activity level. Each reaction was monitored for 5 minutes to confirm that the initial slope of absorbance change was not originated from the noise.

### NADH-quinone reductase activity assay

NADH-quinone reductase (complex I) activity assay was conducted using a reaction mixture containing 50 mM KPi buffer pH 7.4, 1 mM KCN, and 50 μM NADH. The reaction was initiated by introducing 90 μM ubiquinone (UQ_2_) to the reaction mixture. The activity was determined by the oxidation rate of NADH (ϵ = 6.2 mM^-1^cm^-1^) monitoring at 340 nm (JASCO Corporation, spectrophotometer V-760).

### Succinate-quinone reductase activity assay

Succinate-quinone reductase (complex II) activity was determined by monitoring the reduction rate of UQ_2_ (60 μM; ϵ = 12.7 mM^−1^ cm^−1^) at 278 nm (V-760) in the presence of 50 mM KPi buffer pH 7.4, 0.1% (w/v) sucrose monolaurate and 1 mM KCN. The reaction was initiated by adding 10 mM sodium succinate to the reaction mixture.

### NADH-cytochrome c reductase activity assay

NADH-cytochrome c reductase (complex I–III) activity was measured in the presence of 50 mM KPi buffer pH 7.4) containing 2 mM EDTA and 2 mM KCN. 33.3 μM cytochrome c and 10 mM sodium malonate by measuring the reduction rate of cytochrome c at 550 nm (ϵ = 19 mM^-1^cm^-1^) (V-760). This reaction was initiated by the addition of 50 μM NADH to the reaction mixture.

### Succinate-cytochrome c reductase activity assay

Succinate-cytochrome c (complex II–III) activity was measured in the presence of 50 mM KPi buffer pH 7.4 containing 2 mM EDTA, 2 mM KCN, 33.3 μM cytochrome c, and 10 mM sodium malonate by monitoring the reduction rate of cytochrome c at 550 nm (ϵ = 19 mM^-1^cm^-1^) (V-760). This reaction was initiated by adding 10 mM sodium succinate to the reaction mixture.

### NADH-oxidase activity assay

NADH-oxidase (complex I–III–IV) activity was determined in the presence of 50 mM KPi (pH 7.4) as reaction buffer by monitoring the oxidation rate of NADH at 340 nm (ϵ = 6.2 mM^−1^ cm^−1^) (V-760). This reaction was initiated by the addition of 50 μM of NADH to the reaction mixture.

### Ubiquinol-oxidase activity assay

Ubiquinol-oxidase (complex III–IV) activity was determined by monitoring the oxidation rate of ubiquinol-1 (100 μM) at 278 nm (ϵ = 12.7 mM^−1^ cm^−1^) (V-760) in the presence of 50 mM KPi buffer pH 7.4 containing 2 mM EDTA. This reaction was initiated by the addition of 50 μM ubiquinol-1 to the reaction mixture.

### NADH-fumarate reductase activity assay

NADH-fumarate reductase (complex I–II) activity assay was performed under anaerobic conditions, where the reaction contained degassed 50 mM KPi buffer pH 7.4, 50 μM NADH, 100 μg/mL of glucose oxidase, 2 μg/mL of catalase and 10 mM glucose and left for 3 min to achieve anaerobiosis. The reaction was initiated by the addition of 5 mM sodium fumarate as an electron acceptor and the activity was determined by monitoring the oxidation rate of NADH at 340 nm (ϵ = 6.2 mM^−1^ cm^−1^) (SHIMADZU spectrophotometer UV-3000).

### High-resolution clear native electrophoresis for adult mitochondria

Mitochondria were solubilized according to previously described methods (Schägger and von Jagow, 1991) with slight modifications. Briefly, equal volumes of solubilization buffer containing 50 mM Tris-HCl pH 8.0, 4% (w/v) sucrose monolaurate (SML), 40% (v/v) glycerol and 2 mM sodium malonate, and 10 mg/mL mitochondria from the adult *Fasciola* sp. were mixed and incubated on ice for 1 h and centrifuged at 200,000 × g at 4°C for 30 min. The resulting supernatants were subjected to high-resolution clear native electrophoresis (hrCNE) using Invitrogen’s Native PAGE™ 4−16% Bis-Tris gel in duplicate. The cathode buffer for hrCNE, 50 mM Bis-Tris, 50 mM Tricine, pH 6.8 (Invitrogen, Native PAGE™ Running buffer) was supplemented with 0.05% (w/v) sodium deoxycholate (DOC) and 0.02% (w/v) dodecylmaltoside (DDM), and the gel was run in the cold room (4°C). The initial voltage for the gel run was set to 100 V, and after 1 h, the voltage was raised to 250 V. For molecular weight determination, a NativeMark™ Unstained Protein Standard was used as a reference. Adult *A. suum* and bovine mitochondria were used as controls. After hrCNE, both gels were used for visualization, one for assessing complex I through NADH dehydrogenase (NDH) activity staining and the other for complex II through succinate dehydrogenase (SDH) activity staining. Both gels were soaked in 5 mM Tris-HCl buffer, pH 7.4 containing 1.5 mM nitro blue tetrazolium (NBT) salt. Subsequently, 100 µM NADH was added for NDH activity staining, or 10 mM sodium succinate and 100 µg/mL phenazine methosulfate (PMS) were added for SDH activity staining at room temperature (25°C). After 3 min, the reagents were washed with distilled water and the resulting bands were documented.

### Enzyme inhibition assays for adult mitochondria

The inhibition potencies of three typical respiratory chain enzyme inhibitors against mitochondrial complexes of adult *Fasciola* flukes were determined, i.e., each activity assay was repeated as described above in the presence of the inhibitors. The inhibitors were incubated for 2 minutes prior to initiation of enzyme reactions. The inhibitors used were rotenone, atpenin A5, and ascochlorin, which target complexes I, II, and III, respectively (**Figure 1**). The final concentrations of the inhibitors were 0.1 μM, 1 μM, and 10 μM, respectively. Inhibition was calculated as the reduction in the enzyme activity with addition of the compound, average of 3 technical replicates.

### Preparation of newly excysted juveniles

The NEJs excysted from the metacercariae were prepared for *in vitro* assays according to a previously described protocol (Andrews et al., 2022) with slight modifications. Briefly, the metacercariae were washed with double distilled water and soaked in 2.2% (w/v) sodium hypochlorite to remove the outer cyst wall debris. The metacercariae were then washed thrice with PBS. The excystment was carried out by incubating the samples for 3 h at 39°C in a disposable dish sealed with Parafilm (Bemis Flexible Packaging, Wisconsin, USA). The incubation solution consisted of 120 mM NaHCO_3_, 140 mM NaCl, 0.4% (w/v) taurocholic acid, 33 mM l-cysteine, and 50 mM HCl. The resulting NEJs were washed in RPMI-1640 (Sigma-Aldrich, St Louis, MO, USA) and maintained in a 5% CO_2_ incubator (Mini CO_2_ incubator 4020, Asahi Life Science, Saitama, Japan) at 37°C until use. Each *in vitro* assay was initiated on the day of excystment.

### *In vitro* treatment of NEJs by the specific inhibitors

To examine the killing efficacy of the three inhibitors, rotenone, atpenin A5, and ascochlorin, against living *Fasciola* NEJs, the parasites were maintained in the culture medium supplemented with 10 and 100 μM of each inhibitor. Each well received 10 NEJs, and the volume of the culture medium was 2 mL. This medium consisted of RPMI-1640, 10% (v/v) fetal bovine serum (Funakoshi, Tokyo, Japan), penicillin-streptomycin solution mix (at concentrations of 100 U/mL and 100 ng/mL, respectively, from Sigma-Aldrich), 500 ng/mL of amphotericin B (Thermo Fisher Scientific, Massachusetts, USA), and 10 mM HEPES (Dojindo, Kumamoto, Japan). In addition to the three inhibitors, positive (triclabendazole; Tokyo Chemical Industry, Tokyo, Japan) and negative (dimethyl sulfoxide (DMSO); FUJIFILM Wako Pure Chemical, Osaka, Japan) controls were prepared. The parasite cultures were stored in 12-well plates and incubated at 37°C for 7 days. Under aerobic conditions, the plates were placed in a 5% CO_2_ incubator (Mini CO_2_ Incubator 4020) with O_2_ concentration equal to air (approximately 20%). The effect of each inhibitor was evaluated daily. For anaerobic conditions, the plates were placed in an anaerobic container containing an Anaero Pack (Mitsubishi Gas Chemical, Tokyo, Japan), in which the CO_2_ concentration exceeded 15% and O_2_ concentration was maintained at less than 0.1%. The effects of each inhibitor were evaluated on alternate days. Unlike NEJs, adults are not stable in the *in vitro* condition for 7days. Therefore, we could not perform the *in vitro* assays for adults.

### Relative mortality score of NEJs

The mortality scores of NEJs were evaluated under an inverted microscope (IMT, Olympus, Tokyo, Japan) according to the criteria described by Lorsuwannarat et al. (2014), with slight modifications. The scores were as follows: active motility = 3; inactive, but with visible organs = 2; inactive, with dark color and invisible organs (almost dead) = 1; and inactive with tegument disruption = 0 (clearly dead). The mean scores for 10 NEJs within each well were determined. The Mann-Whitney test was employed to evaluate the significance between DMSO and the inhibitors.

### Propidium iodide staining of NEJs

The NEJ viability was evaluated using propidium iodide (PI) staining (Dojindo Laboratories, Kumamoto, Japan). PI emits red fluorescence (λ_ex_ = 530 nm, λ_em_ = 620 nm) by binding to the nucleic acid of dead cells. The mortality of NEJs was objectively determined, with emission scoring 0 (dead cells) and no emission (living cells) scoring 1. The mean score of 10 NEJs per well was calculated using the following procedure. The culture medium in each well was washed thrice with PBS. After filling each well with 2 mL PBS, 2 μL of 1 mg/mL PI was added and incubated for 30 min at 37°C with 5% CO_2_. Finally, the plates were examined under an inverted fluorescence microscope (IX73; Olympus). The Mann-Whitney test was employed to evaluate the significance between DMSO and the inhibitors.

### Analysis of the quinone components of adult and NEJs

Quinone components from adult *Fasciola* flukes and NEJs were analyzed according to a method described previously (Shiobara et al., 2015). A frozen adult *Fasciola* sp. was cut into four pieces and put in BioMasher^®^ II 1.5-mL tubes (Nippi Inc., Tokyo, Japan). Samples were then homogenized in 20 volumes of 2-propanol and placed in a bath sonicator for 2 min. After centrifugation, 4 µL of the supernatant was injected into two analytical columns (Inertsil ODS-HL, 3 µm, 150 × 2.1 i.d., GL Science, Japan), a reduction column (CQ-R, 20 × 2.0 i.d., Shiseido, Japan) at 40°C. The quinones and quinols were detected using an electrochemical detector at 600 mV against Ag/AgCl (NANOAPACE SI-2, Shiseido, Japan). The mobile phase consisted of 50 mM sodium perchlorate in methanol/2-propanol/water (31/65/4, v/v/v) at a flow rate of 0.18 mL/min. The total UQ_10_ content was calculated as the sum of quinone and quinol, and the quinone form alone was observed in RQ_9_ and RQ_10_. Extraction from the NEJs (200 individuals) was initiated by sonication in 2-propanol. Since the quinone content was limited, the extracts were once dried *in vacuo*, then dissolved in 1/10 volume of 2-propanol, and 8 µL was applied to HPLC. Quinone content was determined based on the wet weight of the parasites, and calculations were made using data from three analyses.

## Results

### Preparation of mitochondrial fractions from adult *Fasciola* flukes and characterization of respiratory activities

In this study, 34.8 mg (protein) of the mitochondrial fraction was obtained from 2.0 g (wet weight) of adult *Fasciola* flukes. Then specific enzyme activities involved in the mitochondrial respiratory chain of adult *Fasciola* flukes were examined (**Table 1**). Adult *Fasciola* mitochondrial NADH-quinone reductase (complex I) exhibited a specific activity of 204 nmol/min/mg. The activity of succinate quinone reductase (complex II) was 635 nmol/min/mg. NADH-cytochrome c reductase (complex I–III) activity was 172 nmol/min/mg. Adult *Fasciola* succinate cytochrome c reductase (complex II–III) showed a specific activity of 60.4 nmol/min/mg. NADH-oxidase (complex I–III–IV) activity was found to be 9.34 nmol/min/mg. The activity of ubiquinol-oxidase (complex III–IV) was 7.42 nmol/min/mg. The specific activity of NADH–fumarate reductase (complex I–II) was 68.0 nmol/min/mg, which is higher than that of adult *A. suum* (51.7 nmol/min/mg), whose fumarate respiration mechanism has been well studied (Iwata et al., 2008).

**Table 1:** Specific activities of mitochondrial respiratory chain enzyme in adult *Fasciola* fluke compared with adult *A. suum*.

	Specific activity (nmol/min/mg)	
	*Fasciola* sp.	*A. suum*
NADH-quinone reductase (complex I)	204 ± 2.51	124 ± 4.80
Succinate-quinone reductase (complex II)	635 ± 15.3	248 ± 10.5
NADH-cytochrome c reductase (complex I−III)	172 ± 12.9	29.6 ± 0.77
Succinate-cytochrome c reductase (complex II−III)	60.4 ± 3.17	32.3 ± 0.77
NADH-oxidase (complex I−III−IV)	9.34 ± 0.22	18.8 ± 2.1
Ubiquinol-oxidase (complex III−IV)	7.42 ± 0.97	0.67 ± 0.001
NADH-fumarate reductase (complex I−II)	68.0 ± 5.16	51.7 ± 8.30

All values represent the means of triplicate assays.

Mitochondria of both parasites were prepared according to a previously described protocol for adult *A. suum* mitochondria (Iwata et al., 2008)

### hrCNE for adult mitochondria

**Figure 3:**
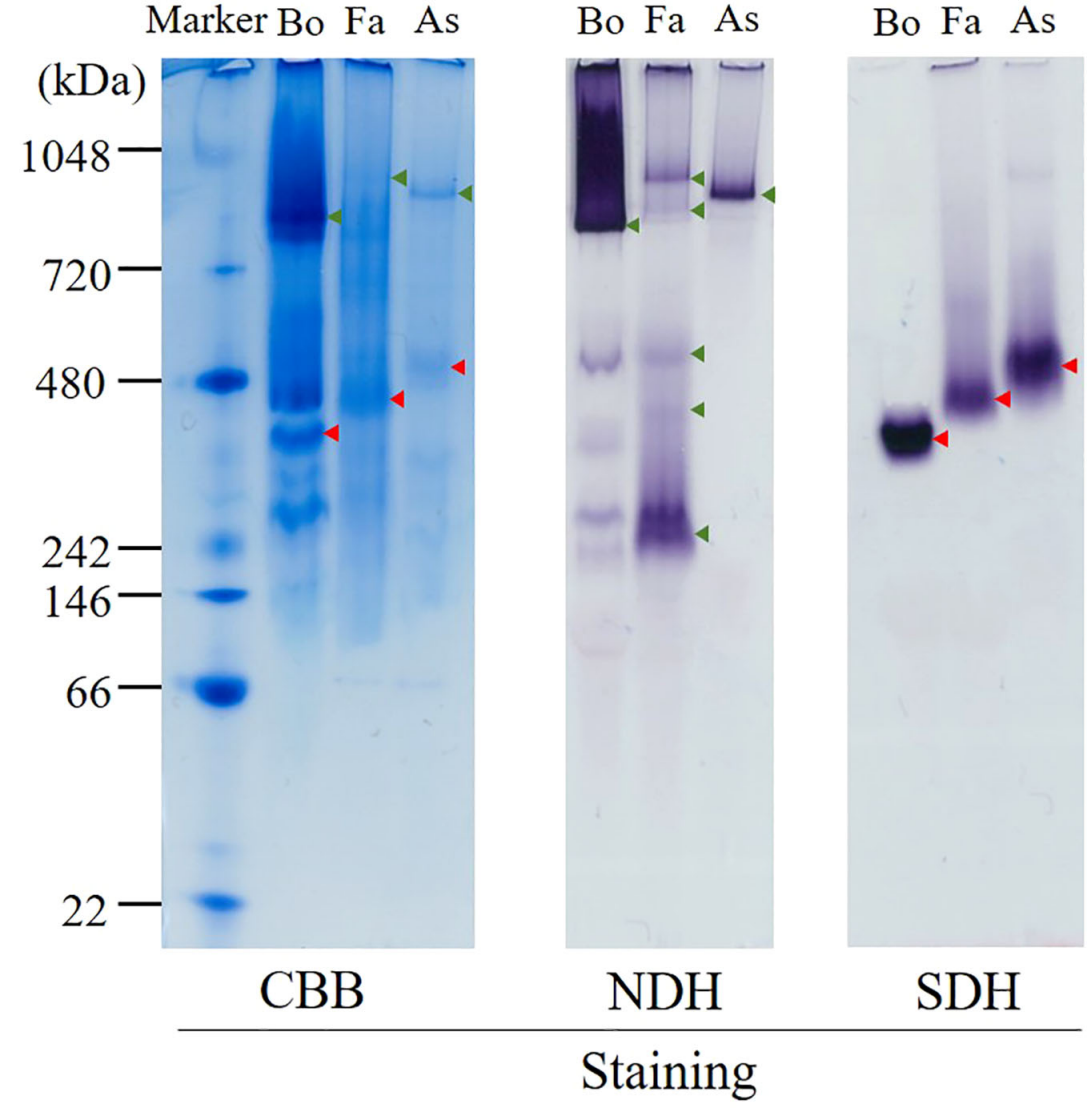
High-resolution clear native electrophoresis (hrCNE) analysis of mitochondria from bovine (Bo), *Fasciola* (Fa), and *Ascaris suum* (As). Marker: NativeMark™ Unstained Protein Standard (Thermo Fisher). From left to right: Coomassie Brilliant Blue (CBB) staining, NADH dehydrogenase (NDH) activity staining that visualizes complex I, succinate dehydrogenase (SDH) activity staining that visualizes complex II. Arrowheads indicate the mitochondrial proteins that are active in NDH (green) and SDH (red) staining.

The hrCNE (**Figure 3**) was used to compare the properties of complexes I and II from *Fasciola* flukes with those of bovine and *A. suum*. *Fasciola* complexes I and II were distinct in size compared to those of bovines and *A. suum*. NDH activity staining, which targeted complex I, revealed that multiple bands were stained in *Fasciola* and bovine mitochondria, but they were different in size. In contrast, only a single band was observed for *A. suum*. Complex II was visualized via SDH activity staining, and the results showed that complex II of *Fasciola* was smaller than that of *A. suum* but larger than that of bovine. These findings indicate that the sizes of complexes I and II in *Fasciola* flukes are unique and differ from those of bovine and *A. suum*.

### Enzyme inhibition assays for adult mitochondria

Specific inhibitors of each enzyme were employed in inhibition assays to investigate the properties of the respiratory chain of adult *Fasciola* flukes. The percentage inhibition of each enzyme in the mitochondrial respiratory chain of adult *Fasciola* flukes are shown in **Table 2**. Rotenone inhibits all activities of complex I. The inhibition of complexes I, I–II, and I–III–IV activities were 91.0%, 94.1%, and 100%, respectively. Similarly, atpenin A5 inhibits complex II-dependent activity. The inhibition at 1 μM were 97.2%, 92.0%, and 90.5% for complexes II, I–II, and II–III, respectively. Again, 1 μM of ascochlorin inhibited complex III-related activities. It inhibited complexes II–III and I–III–IV by 83.9% and 82.6%, respectively. Notably, ascochlorin unexpectedly inhibited complex II-related activity, although it is known to be a specific inhibitor of complex III. The inhibition of ascochlorin was 72.3% and 85.0% against complexes II and I–II, respectively.

**Table 2:** Percentage inhibition of *Fasciola* fluke mitochondrial enzyme complexes by classical inhibitors.

		NADH-quinone reductase (complex I)	Succinate-quinone reductase (complex II)	NADH-cytochrome c reductase (complex I−III)	NADH-oxidase (complex I−III−IV)	Ubiquinol-oxidase (complex III−IV)	NADH-fumaratereductase (complex I−II)
Rotenone	0.1 µM	91.0 ± 1.80	15.0 ± 8.20	73.6 ± 2.90	100 ± 0	10.0 ± 0	94.1 ± 1.50
Atpenin A5	0.1 µM	8.60 ± 9.46	82.8 ± 0.60	44.3 ± 5.40	32.2 ± 31.6	17.1 ± 17.5	79.5 ± 10.5
	1 µM	16.1 ± 14.5	97.2 ± 0.80	90.5 ± 2.20	49.0 ± 20.0	27.3 ± 12.8	92.0 ± 4.93
	10 µM	53.0 ± 1.10	95.0 ± 1.10	99.0 ± 0.50	47.1 ± 24.1	46.5 ± 17.7	97.2 ± 4.68
Ascochlorin	0.1 µM	2.10 ± 10.9	37.6 ± 7.10	78.0 ± 4.63	77.5 ± 9.94	31.9 ± 12.4	79.5 ± 6.94
	1 µM	8.40 ± 2.00	72.3 ± 1.90	83.9 ± 8.01	82.6 ± 9.68	24.9 ± 20.9	85.0 ± 7.07

Inhibition was calculated as the reduction in the enzyme activity with addition of the compound, average of 3 technical replicates.

### *In vitro* treatment of NEJs by the specific inhibitors

**Figure 4:**
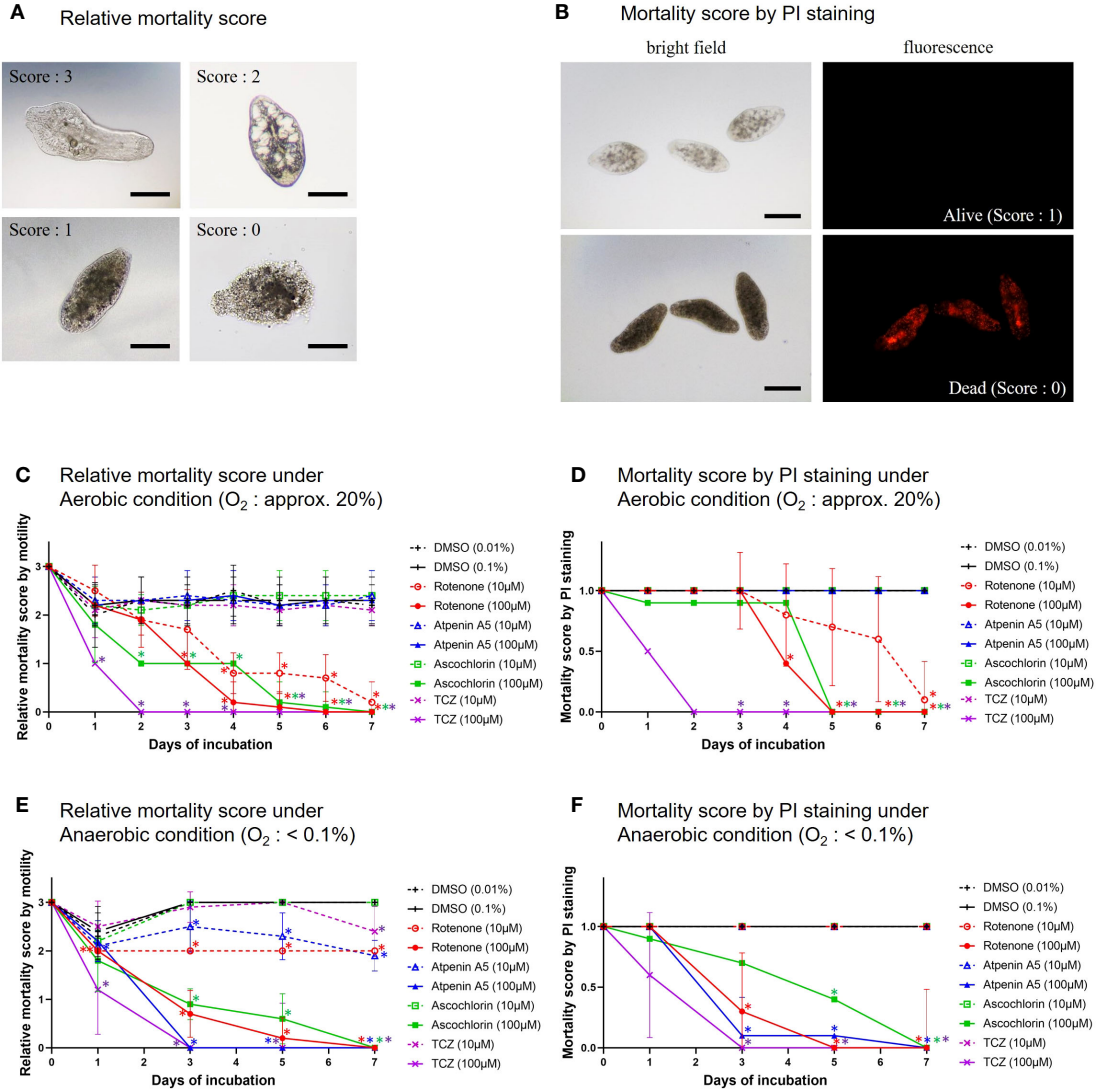
*In vitro* treatment of NEJs by the specific inhibitors. **(A)** The representative images of mortality scores in the *in vitro* assays. 3: NEJs with active movement, 2: Inactive but the anatomical structures of the NEJ were observed, 1: Inactive and the anatomical structures of the NEJ were not clear, 0: The tegument was disrupted (completely dead). Bar: 100 μm. **(B)** The representative images of propidium iodide (PI) staining for NEJs in the *in vitro* assays. PI emits red fluorescence due to the intercalation with DNA in dead cells. Alive = no emission, scoring 1. Dead = with emission, scoring 0. Bar: 100 μm. **(C)** Relative mortality score under aerobic condition. **(D)** Mortality score by PI staining under aerobic condition. **(E)** Relative mortality score under anaerobic condition. **(F)** Mortality score by PI staining under anaerobic condition. **(C)** to **(F)** The mean score ± standard deviations of 10 NEJs per well were plotted for 7days. DMSO (dimethyl sulfoxide): Negative control, 0.01% and 0.1% were for 10 µM and 100 µM of the inhibitors, respectively. Each inhibitor was shown in its unique color. TCZ (triclabendazole): Positive control. The Mann-Whitney test was employed to evaluate the significance between DMSO and the inhibitors. An asterisk in the color of each inhibitor indicates that there is a significant difference compared to DMSO.

To understand which enzyme complexes are essential for juvenile survival, we performed *in vitro* assays to assess the killing efficacy of three typical inhibitors against NEJs (**Figure 4**). Killing efficacy was evaluated using the relative mortality score and PI staining. The results obtained using both methods were consistent with each other. Under aerobic conditions, rotenone was effective at both 10 and 100 μM. Ascochlorin killed NEJs at 100 μM but it was not effective at 10 μM. In contrast, atpenin A5 was ineffective at both concentrations. Under anaerobic conditions, rotenone, ascochlorin, and atpenin A5 at 100 μM killed NEJs. Interestingly, the cytotoxic efficacy of atpenin A5 was observed only under anaerobic conditions.

### Quinone components of adults and NEJs

The quinone components of adults and NEJs were investigated to reveal changes in electron mediators between the two stages. The total UQ_10_, RQ_10_, and RQ_9_ concentrations in adult *Fasciola* fluke were 1.5 ± 0.24, 55 ± 2.9, and 1.4 ± 0.89 pmol/mg of the parasite wet weight, respectively. In contrast, the content of total UQ_10_ in NEJs was 1.4 ± 0.041 pmol/mg and that of RQ_10_ was 1.5 ± 0.53 pmol/mg. The RQ_10_/UQ_10_ ratios were 36.7 and 1.07 for adults and NEJs, respectively. Consequently, RQ_10_ required for fumarate respiration was predominant in adults. In contrast, in addition to RQ_10_, UQ_10_ used in oxygen respiration was detected almost equally in NEJs (**Table 3**).

**Table 3:** Quinone components.

Stage	UQ_10_	RQ_10_	RQ_9_	RQ_10_/UQ_10_
Adult	1.50 ± 0.24	55.0 ± 2.9	1.40 ± 0.89	36.7
NEJ	1.40 ± 0.041	1.50 ± 0.53	ND	1.07

pmol/mg (wet weight), ND: Not detected

## Discussion

In the present study, we analyzed the enzymatic activity of the mitochondrial respiratory system, which is essential for the survival of *Fasciola* flukes. This is the first attempt for the parasite and could serve as a model for Trematoda, as no similar analysis has been performed for other parasitic trematodes to date. High specific activities of complexes I and II were observed in the adult mitochondrial fraction, similar to those of *A.suum* (**Table 1**). In contrast, the activities of complexes III and IV in adult flukes were much lower than those of complexes I and II (**Table 1**), although this feature is reversed in mammalian hosts (Kita et al., 2003). NADH-fumarate reductase, comprising complexes I and II (**Figure 1**), showed high activity in adult *Fasciola* sp., nearly comparable to that of *A. suum* (**Table 1**). Notably, the hrCNE results (**Figure 3**) showed that the molecular sizes of *Fasciola* complexes I and II were different from those of bovine and *A. suum*, suggesting that *Fasciola* flukes have unique respiratory chain enzyme structures. Therefore, a more detailed protein chemical analysis is required to understand the difference of the enzyme at the molecular level. This will provide an insight into the diversity of the enzymes obtained during the evolution. Furthermore, analyses of the quinone components revealed that RQ_10_ was the primary quinone in adults (**Table 3**), supporting the presence of functional NADH-fumarate reductase in adult flukes.

The typical respiratory inhibitors, rotenone, atpenin A5, and ascochlorin, against complexes I, II, and III, respectively, were effective for the respective enzyme activities in adults, as shown in **Table 2**. Rotenone and atpenin A5 strongly inhibit NADH-fumarate reductase. Notably, ascochlorin inhibited complexes II and III. It is worth mentioning that the inhibition of NADH-fumarate reductase by ascochlorin was more pronounced than expected (**Table 2**). A recent study (Enkai et al., 2023) showed that ascofuranone, an analog of ascochlorin, effectively inhibited complex II of *E. multilocularis*. These results suggest that ascochlorin analogs may inhibit complex II of parasitic platyhelminths. Further studies on the structure of each enzyme may reveal the MOA of ascochlorin.

The juvenile stage of *Fasciola* has the highest pathogenicity in its hosts. However, no drugs other than TCZ are available for adults or juveniles (WHO, 2020). Therefore, the validation of drug targets using juvenile stages is important. A previous study demonstrated that aerobically functioning juveniles of *F. hepatica* use the Krebs cycle (hence, SDH) activity (Tielens et al., 1981). The authors suggested that aerobic energy metabolism was gradually replaced by anaerobic energy metabolism during juvenile growth (Tielens et al., 1984). Furthermore, they revealed that the energy metabolism of adult liver flukes was almost exclusively dependent on malate dismutation (Tielens et al., 1984) suggesting fumarate respiration. Subsequent studies support this idea, as changes in UQ and RQ levels were observed during the development from metacercariae to adults in *F. hepatica* (Van Hellemond et al., 1995). The results of this study revealed nearly equivalent amounts of RQ_10_ and UQ_10_ in NEJs (RQ_10_/UQ_10_: 1.07) (**Table 3**). We investigated the respiratory enzymes essential for NEJs *in vitro* to characterize the respiratory chains of NEJs under both aerobic and anaerobic conditions (**Figure 4**). This analysis is important because of the changes in environmental oxygen concentration experienced during the juvenile stage, as illustrated in **Figure 2**.

Lorsuwannarat et al. (2014) used a mortality score to evaluate the efficiency of chemicals against NEJs in an *in vitro* assay. However, the mortality scores tend to be subjective and not fully reliable. Therefore, in this study, we addressed this challenge by utilizing PI staining. The results obtained using both the mortality score and PI staining were well-aligned (**Figure 4**), confirming the reliability of the mortality scores. Under aerobic conditions, the inhibition of complexes I and III by rotenone and ascochlorin, respectively, was lethal to NEJs (**Figure 4**). This indicates that NADH-oxidase activity comprises complexes I and III functions in the respiratory chain of NEJs under high oxygen conditions. In contrast, atpenin A5, the most potent complex II inhibitor, did not kill NEJs under aerobic conditions (**Figure 4**), indicating that the tricarboxylic acid (TCA) cycle with complex II is not essential for the survival of NEJs under aerobic conditions. However, under anaerobic conditions, NEJs are highly dependent on complex II, which accounts for the assay result that atpenin A5 effectively killed NEJs (**Figure 4**). This indicates that complex II, which is involved in fumarate respiration (**Figure 1**), is indispensable for NEJ survival under low-oxygen conditions. The inhibition of complex I by rotenone also affected fumarate respiration and killed NEJs (**Figure 4**). An unexpected effect of ascochlorin on NEJs under the anaerobic conditions (**Figure 4**) was probably due to its inhibitory activity against complex II (72.3%) (**Table 2**) (Enkai et al., 2023). The present study revealed that NEJs can use both oxygen and fumarate respiration (**Figure 1**), depending on oxygen availability. We find that the biochemistry of respiratory complexes of *Fasiocla* flukes are developmentally regulated. NEJs can use both oxygen and fumarate respiration (**Figure 1**), depending on oxygen availability, while adults depend solely on fumarate respiration. We suggest that this developmental shift may be related to the varying oxygen constraints in the vertebrate host. In the *Fasciola* life cycle (**Figure 2**), metacercariae are stimulated in the stomach and hatched in the duodenum (O_2_ ~10%), then experience progressively greater hypoxia during migration to the liver (O_2_ ~5%) where they mature to adults (Mas-Bargues et al., 2019). Owing to the low oxygen concentration in the host, fumarate respiration is essential for the survival of the parasite and is a promising drug target not only for adults but also for NEJs.

In conclusion, we demonstrated that both oxygen and fumarate respiration can be differentially utilized by NEJs, depending on the oxygen availability in the environment, according to the migration route within the host (**Figure 2**). We have shown the active hybrid respiration in the helminths, including *A. suum* (Iwata et al., 2008) and *E. multirocularis* (Enkai et al., 2023), whose adult habitats were low-oxygen conditions, and this is a common adaptation strategy of the parasites. As shown by the hrCNE analysis in the present study, it may be a diverse property acquired by each parasite as it evolves and adapts to its host environment, suggesting that fumarate respiration has evolved independently in each of the parasites. Fumarate respiration is a promising drug target for *Fasciola* flukes because it plays an essential role in both adults and NEJs while remaining inactive in mammalian hosts (Kita et al., 2003). However, because NEJs have active oxygen respiration, ideally, we need to identify drug candidates that can inhibit the parasite’s aerobic and anaerobic respiration. Thus, further research is warranted.

## Data availability statement

The original contributions presented in the study are included in the article/supplementary material. Further inquiries can be directed to the corresponding authors.

## Ethics statement

The animal study was approved by Institutional Animal Care and Use Committee of Iwate University. The study was conducted in accordance with the local legislation and institutional requirements.

## Author contributions

AT: Writing – original draft. DI: Funding acquisition, Methodology, Writing – review & editing. KS: Methodology, Writing – review & editing. KM: Methodology, Writing – review & editing. FZ:. KK: Supervision, Writing – review & editing. MI-S: Funding acquisition, Writing – original draft.
